# Supplementary material for: High-Dose Vitamin D Supplementation Significantly Affects the Placental Transcriptome
Source: Nutrients. 2023 Dec 7;15(24):5032. doi: 10.3390/nu15245032 (PMC10745524; doi:10.3390/nu15245032)
Supplement: Supplementary file 1 [file nutrients-15-05032-s001.zip › nutrients-2729067-supplementary.pdf]

Supplementary

Table S1: Differentially expressed genes following correction of a false discovery rate of 10%

Gene	Protein	Function*	Log2FC	Adjusted p-value
**Vitamin D insufficient/deficient vs vitamin D sufficient in early pregnancy**				
C2	Complement C2	Part of the complement system. Deficiency in C2 complement has been associated to certain autoimmune diseases	2.69	0.00178
XIST	RNA gene, non-protein coding	Involved in X inactivation	2.09	0.00732
SKIC2	Superkiller complex protein 2	A DEAD box protein** which is a putative RNA helicase. Involved in a number of cellular processes including alterations of RNA secondary structure like initiation of translation, nuclear and mitochondrial splicing and ribosome and spliceosome assembly. May be involved in antiviral activity by blocking translation of poly(A) deficient mRNAs	2.44	0.0176
SQSTM1	Sequestosome-1	Binds ubiquitin and regulates activation of the nuclear factor kappa-B (NF-kB) signaling pathway. Mutations in this gene are associated to Paget disease of the bone.	1.29	0.0191
RNU1-4	RNA gene, non-protein coding	Is involved in the pathway for Spliceosomal Splicing Cycle.	1.38	0.0824
PTH	Parathyroid hormone	Regulates blood calcium and phosphate. Stimulates calcium release from bones and inhibit calcium excretion in the kidneys. Stimulates the CYP27B1 activity resulting in increased 1,25(OH)$_2$D	1.66	0.0824
**90µg vs 10µg for all samples (n=70) with correction for offspring sex**				
EIF3K	Eukaryotic translation initiation factor 3 subunit K	Component of the eukaryotic translation initiation factor 3 complex which is necessary for initiation of protein synthesis especially translation of a subset of mRNAs involved in cell proliferation, including cell cycling, differentiation, and apoptosis.	1.70	0.0132

TDRD12	Putative ATP-dependent RNA helicase TDRD12	Predicted to enable ATP binding activity; RNA helicase activity; and nucleic acid binding activity. Involved in gamete generation, gene silencing by RNA, and piRNA metabolic process	2.51	0.0403
JPH1	Junctophilin-1	Component of junctional complexes. Contribute to junctional complexes between the plasma membrane and endoplasmic/sarcoplasmic reticulum in skeletal muscle cells	-1.08	0.0429
**90μg vs 10μg for placentas from pregnancies with boys (n=32)**				
COX5B	Cytochrome c oxidase subunit 5B, mitochondrial	Subunit 5B of the Cytochrome C oxidase which is the terminal enzyme of the mitochondrial respiratory chain	3.52	0.00316
NLRP2	NACHT, LRR and PYD domains-containing protein 2	Member of the nucleotide-binding and leucine-rich repeat receptor family which are thought to be involved in regulation of immune responses	2.76	0.0461
LRRC15	Leucine-rich repeat-containing protein 15	Facilitates collagen binding activity, fibronectin binding activity and laminin binding activity	-3.88	0.0461
TDRD12	Putative ATP-dependent RNA helicase TDRD12	Predicted to enable ATP binding activity; RNA helicase activity; and nucleic acid binding activity. Involved in gamete generation, gene silencing by RNA, and piRNA metabolic process	-3.14	0.0978
**90μg vs 10μg for placentas from pregnancies with girls (n=38)**				
DDR1	Epithelial discoidin domain-containing receptor 1	Receptor tyrosine kinase involved in the regulation of cell growth, differentiation, and metabolism	1.90	0.0915

* Information subtracted from https://www.genecards.org/

** DEAD box proteins are proteins characterized by the conserved motif Asp-Glu-Ala-Asp (DEAD)

NACHT: nucleotide-binding and oligomerization domain

LRR: Leucine-rich repeats

PYD: pyrin effector domain
